# F‐Type Pyocin Versus Phage λ Tail: Conserved Hub, Divergent Fibers

**DOI:** 10.1002/advs.77063

**Published:** 2026-08-10

**Authors:** Zhiwei Gu, Yufan Xie, Lanxin Wang, Luyan Ma, Xiaofei Ge, Jiawei Wang

**Affiliations:** ^1^ State Key Laboratory of Membrane Biology Beijing Frontier Research Center for Biological Structure School of Life Sciences Tsinghua University Beijing P. R. China; ^2^ State Key Laboratory of Microbial Diversity and Innovative Utilization Institute of Microbiology Chinese Academy of Sciences Beijing P. R. China; ^3^ University of Chinese Academy of Sciences Beijing P. R. China; ^4^ Health and Wellness City University of Macau Macau P. R. China

**Keywords:** bacteriocin, lipopolysaccharide (LPS) recognition, phage tail‐like bacteriocin, *pseudomonas aeruginosa*, PTLB, pyocin, tailocin

## Abstract

Bacteriocins are ribosomally synthesized antimicrobial peptides or proteins that offer an alternative to conventional antibiotics against multidrug‐resistant pathogens. Phage tail‐like bacteriocins (tailocins) are classified into rigid R‐type and flexible F‐type variants. While R‐type pyocins from *Pseudomonas aeruginosa* are well‐characterized, F‐type pyocins remain poorly understood, especially with respect to the molecular mechanisms for their Gram‐negative bactericidal activity. Here, we report cryo‐electron microscopy structures of the F‐type pyocin from *P. aeruginosa* ATCC 15442 at 2.29–3.26 Å resolution, encompassing three modular components: the tail cap, tail tip, and tail fiber. Structural comparisons with bacteriophage λ reveal a conserved tail tip architecture, including the baseplate hub proteins, distal tail protein, tail assembly protein, and tape measure protein. Unexpectedly, we identify three trimeric side fibers that attach not to the distal tail protein, as in canonical systems, but to the α‐helical shaft of the central fiber, indicating a previously unrecognized attachment mode. The receptor‐binding domain of the side fiber shares structural similarity with LPS‐recognizing domains of R‐type pyocins. Together, these results define the structural basis of F‐type pyocin assembly and host recognition, reveal conserved and unique features relative to phage λ, and provide a framework for engineering tailocins as precision antimicrobials against drug‐resistant *P. aeruginosa*.

## Graphical abstract

Near‐atomic cryo‐EM structures of a Gram‐negative F‐type pyocin reveal a conserved tail tip architecture shared with bacteriophage λ together with a previously unrecognized side‐fiber attachment mode. These findings provide new insight into tailocin assembly and host recognition and establish a structural framework for engineering precision antimicrobials against drug‐resistant bacteria.

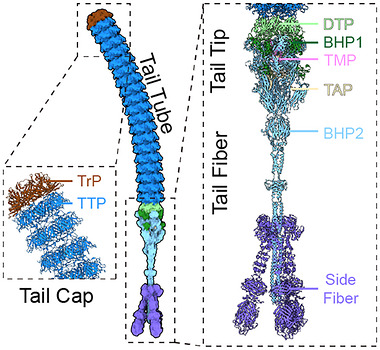

## 1 Introduction

Bacteriocins are antibacterial proteins or peptides produced by bacteria that inhibit the growth of closely related strains, often at low concentrations [1]. They encompass diverse molecular architectures, including small peptides, soluble proteins, and large proteinaceous assemblies [2, 3]. Phage tail‐like bacteriocins (PTLBs), also known as tailocins, form a distinct subset of high‐molecular‐weight bacteriocins with architectures closely resembling bacteriophage tails [4, 5, 6].

PTLBs induce target cell death following specific receptor binding and function as a form of microbial altruism, in which producer cells undergo autolysis to protect the bacterial population [7, 8]. They are broadly classified into two subtypes: rigid R‐type and flexible F‐type [9, 10]. R‐type PTLBs consist of stiff, contractile nanotubes analogous to myophage tails [11, 12], whereas F‐type variants form flexible, non‐contractile rod‐like structures resembling siphovirus tails [10]. PTLBs are typically named after their producing organisms, with representative examples including diffocin from *Clostridium difficile* [13], monocin from *Listeria monocytogenes* [14, 15, 16], and pyocin from *Pseudomonas aeruginosa* [17].

*P. aeruginosa* is a major opportunistic pathogen responsible for severe multidrug‐resistant infections, particularly in immunocompromised individuals and patients with cystic fibrosis [18]. The increasing prevalence of drug‐resistant *P. aeruginosa* infections underscores the need for alternative antimicrobial strategies [19]. Certain *P. aeruginosa* strains produce exclusively R‐type pyocins, others only F‐type pyocins, while a subset synthesizes both variants simultaneously [10]. High‐resolution structures of R‐type PTLBs from diverse bacteria have been determined in both pre‐ and post‐contraction states, providing a detailed understanding of their killing mechanisms [20, 21]. These studies have enabled mechanistic insights into R‐type pyocins from Gram‐negative bacteria, supporting their development as potential antibiotic alternatives.

In contrast, F‐type pyocins remain poorly characterized. To date, the only atomic structure of an F‐type PTLB is monocin from the Gram‐positive bacterium *L. monocytogenes*, which contains a single membrane [14]. *P. aeruginosa*, however, is a Gram‐negative bacterium. Whether F‐type pyocins overcome the additional outer membrane barrier of Gram‐negative bacteria through mechanisms similar to or distinct from those of monocin remains unknown. This gap highlights the importance of structural and mechanistic studies of F‐type pyocins. F‐type pyocins are divided into multiple subgroups (F1–F2 and F4–F12), each of which targets *P. aeruginosa* with several certain serotypes [10]. While the core structural genes are highly conserved across these variants, prominent sequence heterogeneity is exclusively restricted to the genomic regions encoding target‐specificity determinants. For this reason, structural characterization of a single representative subtype can uncover the conserved architectural features and shared mode of action common to all F‐type pyocins.

Here, we report the cryo‐electron microscopy (cryo‐EM) structure of endogenous F2‐type pyocin isolated natively from *P. aeruginosa* strain ATCC 15442, which displays bactericidal activity against multiple *P. aeruginosa* strains belonging to serotypes O3, O6, and O13/14 [10]. Given the shared infection context in Gram‐negative bacteria, we perform structural comparisons with bacteriophage λ tails to identify conserved and divergent features. Based on these analyses and established models of phage λ tail function, we further propose a structural framework for host receptor recognition by F‐type pyocins.

## 2 Results

### 2.1 Structure of F‐type Pyocin

**FIGURE 1:**
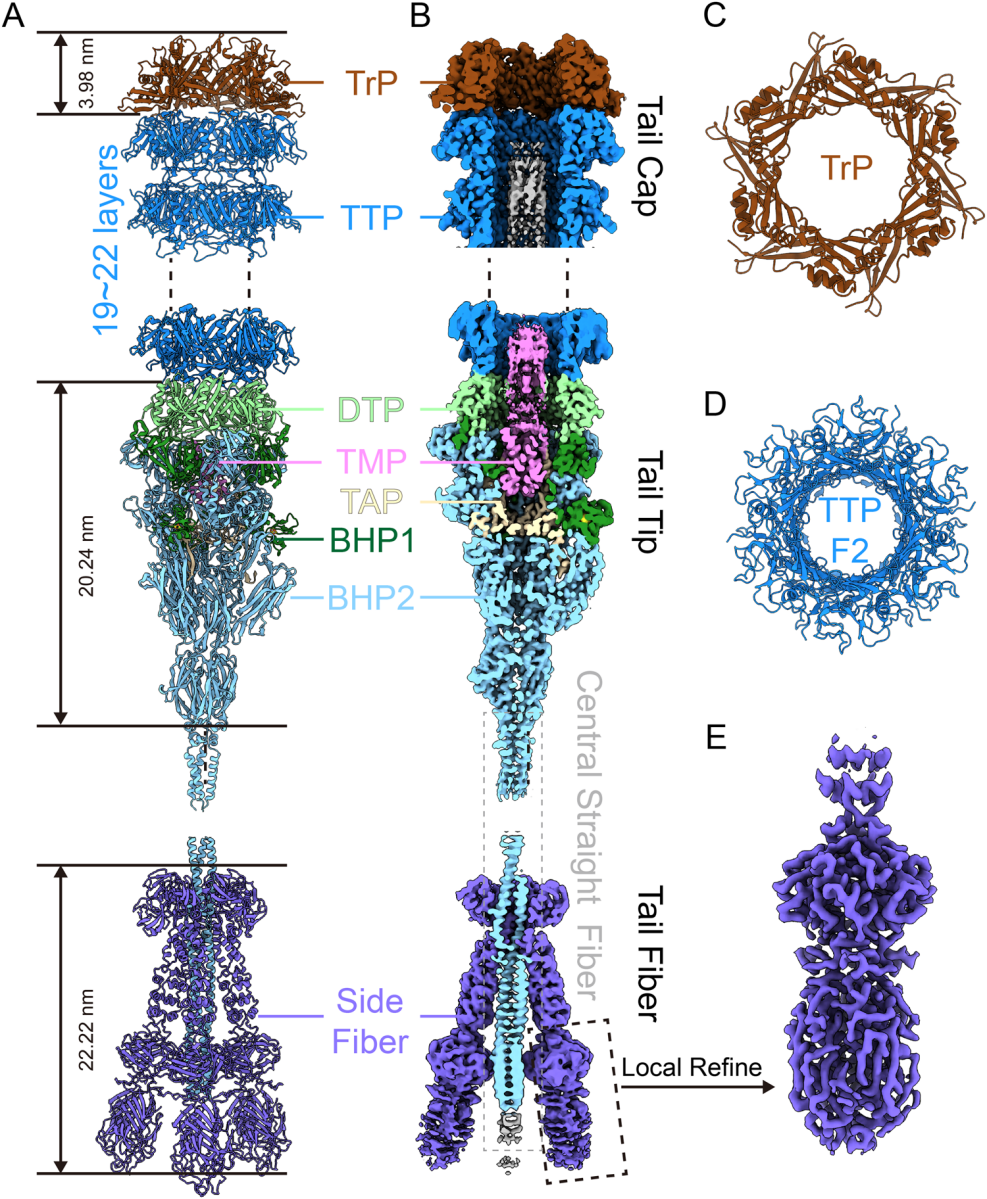
Overall structure of the F‐type pyocin. (A) Atomic model of the F‐type pyocin, showing its modular architecture. (B) Corresponding cross‐sectional views of the cryo‐EM maps, highlighting internal structural features. (C) Top view of the terminator protein (TrP) hexamer. (D) Top view of the tail tube protein hexamer. (E) Locally refined high‐resolution cryo‐EM map of the side fiber.

*P. aeruginosa* (ATCC 15442) strains that exclusively produce F‐type pyocins were used for F2‐type pyocin production. Following anion‐exchange chromatography, peak fractions were concentrated for cryo‐EM analysis (Figure S1). In contrast to the bacteriophage λ tail [22, 23] and monocin [14], which is rigidified by inter‐layer disulfide bonds within the tail tube, the F‐type pyocin tail tube adopts a highly flexible conformation (see micrograph in Figure S1A). This intrinsic flexibility precludes high‐resolution reconstruction of the intact particle. To overcome this limitation, pyocin particles were segmented into three modules for independent reconstruction: the tail cap, tail tip, and tail fiber (Figure 1A,B). These modules were resolved at 2.55, 2.29, and 3.26 Å (Table S1), respectively, enabling reliable side‐chain assignment and atomic modelling for most of the F2‐type pyocin structure (Table S2).

The F2‐type pyocin gene cluster comprises 18 genes (*pyoF1*–*pyoF18*), including duplicated gene pairs with sequence divergence (*pyoF13*–*pyoF15* and *pyoF16*–*pyoF18*). Overall, these genes recapitulate the canonical conserved architecture of siphoviruses tail assemblies. In this work, we adopt the conventional nomenclature and corresponding abbreviations established for phage tail proteins to annotate each structural component (Table S2). In line with other non‐contractile phage tails, the elongated tubular F2‐type pyocin is sealed at the tail tip distant end by the tail terminator protein (TrP), with a height of 3.98 nm. Notably, TrP is encoded at a distinct genomic locus [10], which was previously annotated as *alpD* and represented a gene with uncharacterized product within the Alp operon [24]. The Alp operon has been verified to trigger cellular lysis in response to UV‐induced DNA damage and is co‐expressed alongside the Prt operon responsible for pyocin production [25]. Heterologous expression of the full gene cluster in *Escherichia coli* produced pyocin particles of variable, effectively unbounded length that retained bactericidal activity. In contrast, replacement of *pyoF1* with *alpD/TrP* yielded particles with defined length and preserved activity (Figure S2).

The distal end of the tail tube connects to the tail tip complex, which spans 20.24 nm and comprises five proteins: distal tail protein (DTP/PyoF6), baseplate hub proteins BHP1 (PyoF7) and BHP2 (PyoF10, resolved up to residue 819), tail assembly protein (TAP/PyoF9), and tape measure protein (TMP/PyoF5) (Figure 1A). As in bacteriophage λ, the C‐terminus of BHP1 extends to form the central straight fiber (CSF). In phage λ, the CSF corresponds to residues 808–998 (excluding the receptor‐binding domain). By contrast, the homologous region in F‐type pyocin covers residues 819–1204, demonstrating that its CSF is markedly longer. A prominent feature of the F‐type pyocin CSF is an extended α‐helical shaft (AHS), which contains a distinct insertion domain located 5.7 nm from the distal end of the tail tip (Figure S3). Apart from this insertion, the AHS forms a canonical three‐stranded coiled‐coil (Figure 1A). Unexpectedly, three trimeric lateral fibers were observed to associate with this exceptionally long AHS region.

The tail tube consists of 19–22 stacked rings of hexameric tail tube protein (TTP/PyoF2) (Figure S4). TrP (Figure 1C), TTP (Figure 1D), DTP, and the DTP‐interacting domains of BHP1 and BHP2 all adopt the conserved tail tube fold characteristic of long‐tailed phages [26]. Local refinement of the side fiber density yielded a high‐resolution map with well‐resolved side chains, allowing unambiguous assignment of the side fiber protein as PyoF13 (Figure 1E).

### 2.2 Tail Tip Region: Comparison With Phage λ

**FIGURE 2:**
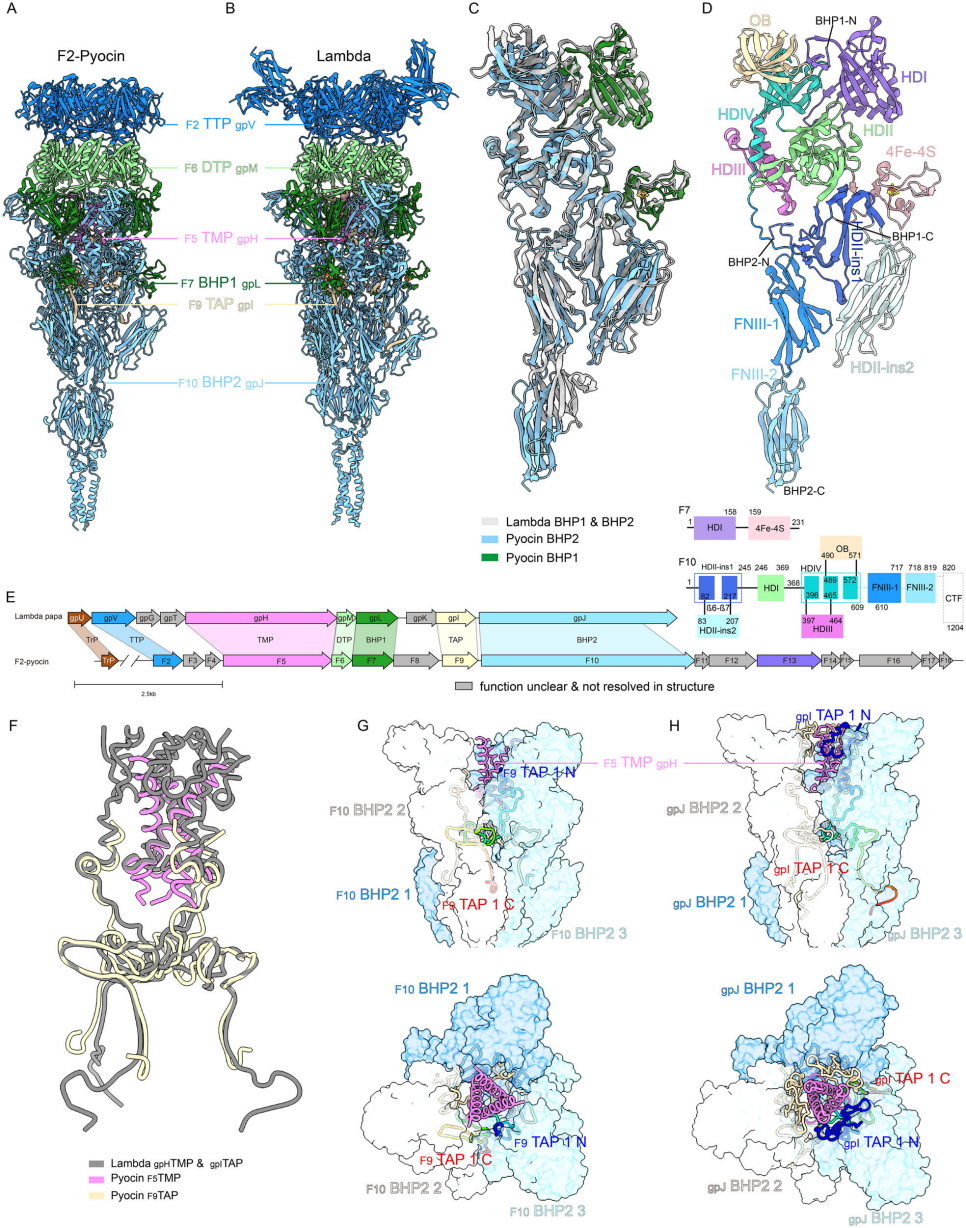
Structural similarity in the tail tip region between the F‐type pyocin and the bacteriophage λ tail. (A) Overall architecture of the F‐pyocin tail tip complex. (B) Corresponding tail tip complex of bacteriophage λ for structural comparison. (C) Superimposition of the baseplate hub regions from the F‐type pyocin and bacteriophage λ, demonstrating structural conservation. (D) Color‐coded representation of the different subdomains within the baseplate hub region of the F‐type pyocin, with labeled N‐ and C‐termini. Corresponding domain boundaries are displayed below following the identical color coding. (E) Schematic showing the correspondence in gene organization between the F‐type pyocin and the bacteriophage λ tail. (F) Comparison of the trimeric TAPs and TMPs between the F‐type pyocin and bacteriophage λ. (G) TAP and TMP of the F‐type pyocin. (H) TAP and TMP of the bacteriophage λ tail.

Structural analysis shows that all major components of the F‐type pyocin tail tip have clear structural homologs in bacteriophage λ (Figure 2A–C). The TTP (PyoF2) lacks the C‐terminal Ig‐like β‐sandwich domain present in phage λ gpV, which in phage λ forms homodimers that project outward from the tail tube and orient perpendicular to the tube axis [22]. DTP (PyoF6) is positioned immediately below the distal tail tube ring and assembles into a hexameric ring that extends the tail tube scaffold (Figure S5). Positioned below the DTP at the distal end of the tail tube is the tail tip complex, also termed the baseplate hub protein (BHP). Analogous to its homolog in phage λ, it mediates the tail tube's symmetry switch from sixfold to threefold [27, 28]. BHPs share a conserved core architecture known as the hub domain, which can be divided into four subdomains (HDI–HDIV; Figure 2D). In both F‐type pyocin and phage λ, the tail tip complex comprises two paralogous hub proteins, BHP1 and BHP2, which assemble into a trimeric complex beneath the DTP ring. BHP1 and BHP2 are multi‐domain proteins with distinct structural roles. The N‐terminal region of BHP1 corresponds to HDI and is followed by a 4Fe–4S cluster‐binding domain [29]. The central region of BHP2 functions as HDIV. Both HDI and HDIV contain a tail tube fold that supports pseudo‐sixfold symmetry. Compared with the canonical tail tube fold, one loop in HDIV is replaced by the HDIII subdomain, which introduces local structural rearrangement. HDIV also includes an oligonucleotide/oligosaccharide‐binding domain (OB domain) within a surface loop. Its N‐terminal arm extends toward HDII and connects to two tandem Ig‐like insertions (HDII‐ins‐1 and HDII‐ins‐2), with HDII‐ins‐2 embedded within HDII‐ins‐1. Together, HDII, HDIII, and HDII‐ins‐1 form a distal module that constricts the tail tube lumen. A flexible linker from the BHP1 originates at the HDIV C‐terminus, traverses the BHP surface, and extends toward the distal tip. Two fibronectin type III domains mediate closure of the tube, followed by the AHS, which forms a compact three‐helix bundle. Overall, the architecture is conserved between F‐type pyocin and phage λ (Figure 2E).

Only the C‐terminal region of TAP could be modeled (Figure 2F). TAP forms a trimer that projects into the central cavity, sealing the tail tube and preventing premature leakage. Although the trimeric arrangement is conserved between F‐type pyocin and phage λ, their monomer conformations differ. In F‐type pyocin, each TAP subunit extends toward a neighboring BHP2 protomer (Figure 2G), whereas in phage λ each subunit interacts with the same BHP2 protomer (Figure 2H). TMP acts as the primary determinant of tail length [30]. Three TMP molecules assemble into a coiled‐coil superhelix that stabilizes the tail and interacts with TAP (Figure 2F).

### 2.3 Side Fiber Attachment to the AHS

The high‐resolution cryo‐EM map enabled unambiguous assignment of the side fiber protein as PyoF13 (Figure 1E). PyoF14 and PyoF15 are likely to function as assembly chaperones [31]. Three distinct structural architectures of the distal tail protein (DTP) or tail tip middle proteins (TTMPs) and its associated side fiber apparatus have been characterized across phages and phage tail‐like bacteriocins (Figure S6). Monocin exemplifies one configuration, where DTP harbors a C‐terminal galectin‐like β‐sandwich domain, with the interdomain loop serving as the side fiber attachment interface [14]. Bacteriophage Ur‐λ exhibits a divergent organization: its DTP is composed exclusively of a tail tube fold, and side fibers bearing N‐terminal Ig‐like domains oligomerize into dodecameric ring‐like assemblies encircling the DTP [32]. T5‐like phages adopt a more elaborate layout, with an additional ring‐shaped trimer of TTMPs interposed between the DTP and the tail tip protein [33]. Anchor proteins carrying Ig‐like domains assemble into a dodecameric collar surrounding the TTMP, a structural feature that mediates and stabilizes side fiber anchoring.

**FIGURE 3:**
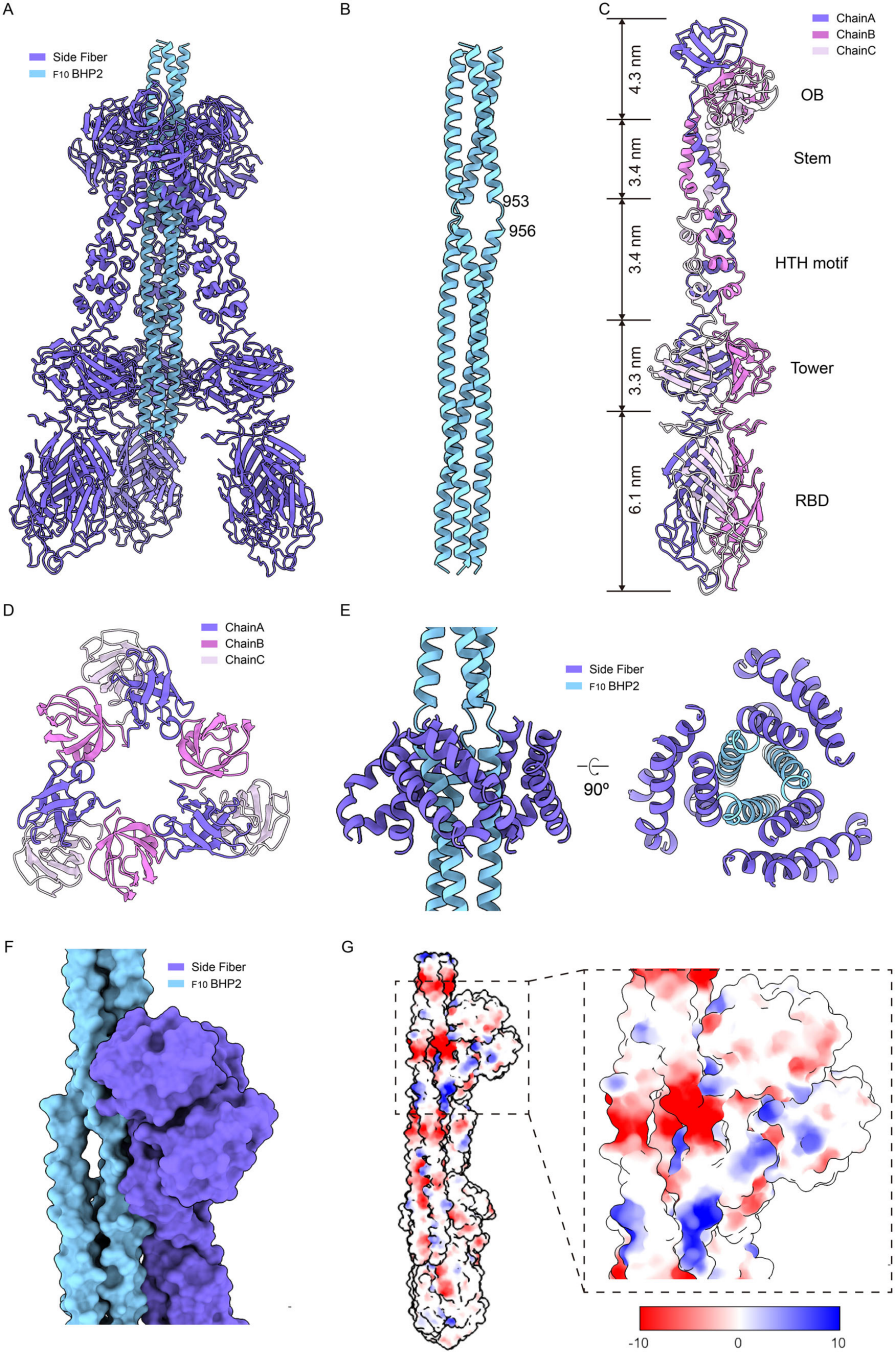
Side fiber region of the F‐type pyocin. (A) Overall structure of the AHS‐side fiber region. (B) Coiled‐coil helical bundle of the AHS. (C) Subdomains of the side fiber. (D) Oligomer formed by the OB‐like domains of the side fiber. (E) Interaction between the stem domains of the side fiber and the AHS. (F) Surface rendering visualizing intermolecular contacts at the side fiber‐AHS interface. (G) Electrostatic potential surface mapping of the side fiber‐AHS interaction interface.

In contrast, three PyoF13 trimers are anchored to the AHS of the central fiber (Figure 3A). The AHS forms a three‐stranded coiled‐coil, and residues 953–956 introduce a structural kink due to G955 (Figure 3B), which serves as the attachment site. Each PyoF13 trimer comprises five domains: an oligonucleotide/oligosaccharide‐binding domain (OB), a stem region, a helix‐turn‐helix motif (HTH), a tower domain, and a receptor‐binding domain (RBD) (Figure 3C). The OB domain is structurally similar to the decorating domain of bacteriophage T5 DTP (Dali Z‐score 6.7; RMSD 2.4 Å; Figure S7) [34]. The helix‐turn‐helix motif resembles that of bacteriophage phi29 head fibers (Z‐score 3.2; RMSD 2.1 Å; Figure S7) [35]. The receptor‐binding domain closely matches that of R‐type pyocin side fibers (Z‐score 11.4; RMSD 2.5 Å; Figure S7) [36], consistent with a role in LPS recognition. The OB domains mediate AHS attachment in a manner analogous to Ig‐like side fiber assemblies. Two subunits from each trimer form a pseudo‐sixfold ring, while the third subunit remains peripheral (Figure 3D). In addition, the stem helices insert into grooves on the AHS surface, forming a six‐helix bundle that further stabilizes the interaction (Figure 3E). The kink domain of AHS carries negative charge, whereas the stem region of the lateral side fiber is positively charged. Their electrostatic complementarity drives mutual interaction (Figure 3F,G).

## 3 Discussion

In this study, we determined near‐atomic structures of the F2‐type pyocin from *P. aeruginosa*. This provides the first high‐resolution structural analysis of an F‐type pyocin from a Gram‐negative bacterium and reveals both conserved and distinct architectural features relevant to tailocin function.

The F2‐pyocin gene cluster contains 18 genes (*pyoF1–pyoF18*). Except for *pyoF1*, its organization shows a clear correspondence to the bacteriophage λ tail gene cluster from *gpV* to *gpJ*, based on gene order and structural assignment (Figure 2E) [28]. Guided by this correspondence and the established phage λ assembly pathway, *pyoF3* and *pyoF4* are likely to function as chaperones in tail tube assembly, analogous to *gpG* and *gpT* [37]. Consistent with this comparison, *pyoF8* (assigned as the *gpK* counterpart) was not resolved, and its function remains unclear. The tail terminator protein (TrP) is encoded outside the main cluster. When the full cluster was expressed in *E. coli*, the particles showed uncontrolled length but retained bactericidal activity. Replacing *pyoF1* with *TrP* restored defined tail length and full activity, indicating a role in length control.

Because of structural flexibility, PyoF10 was resolved only up to the α‐helical shaft (AHS). AlphaFold [38] predicts that the C‐terminus of PyoF10 adopts a β‐prism fold resembling the CSF of phage λ [22], but lacks a canonical receptor‐binding domain (Figure S8A,B). This RBD is likely encoded by one or more proteins within this gene cluster that were not resolved in our cryo‐EM structure. Such an organization is not unprecedented, as the CSF and its receptor‐binding domain are encoded by separate genes in bacteriophage T5 [39]. Consistent with this possibility, AlphaFold predictions indicate that PyoF11 and/or PyoF12 are the most likely candidates for the distal receptor‐binding module. PyoF11 contains one Ig‐like domain (Figure S8C), whereas PyoF12 contains two (Figure S8D), supporting their potential role in receptor recognition [40]. However, this assignment is based on structural prediction and awaits experimental validation. We propose that receptor binding induces rearrangement of these Ig‐like domains and the β‐sheet network in the lower CSF (Figure S8E), which propagates to the AHS and upper CSF regions, leading to reorientation of FNIII domains, rotation of the HDII–HDIII module, and opening of the tail tube. This model is consistent with mechanisms proposed for phage λ and related systems [39, 41].

A notable feature is that three PyoF13 side fiber trimers attach to the AHS rather than to the distal tail protein, as seen in previously characterized systems. This represents a distinct mode of side fiber attachment. If the AHS undergoes a structural transition upon receptor engagement, the side fibers would likely need to dissociate to accommodate this change.

No density signals assignable to PyoF14 and PyoF15 were detected in our reconstructed electron density maps. Knockout‐complementation experiments from other groups have validated that *pyoF14* and *pyoF15* are indispensable genes for sustaining the bactericidal activity of F‐type pyocin [10, 31]. In addition, *pyoF13, pyoF14* and *pyoF15* are functionally coupled and constitute an integrated functional module. Within this module, deletion of an upstream gene fails to be rescued by complementation of the upstream gene alone; concurrent complementation of both upstream and downstream genes is mandatory to restore normal function [10]. We tentatively designate PyoF14 and PyoF15 as side fiber‐associated proteins (Table S2). Gene products of *pyoF16–F18* also remain unresolved, and these proteins are homologs of *pyoF13–F15*.

In summary, we define the near‐complete structure of the F2‐type pyocin and identify an unexpected mode of side fiber attachment to the AHS. The tail tip architecture is highly conserved with bacteriophage λ, supporting a phage‐derived origin. The similarity between the proposed receptor‐binding region and LPS‐binding domains of R‐type pyocins suggests a basis for target specificity. These results provide a structural framework for understanding F‐type pyocin function and support future engineering of tailocins as antimicrobials.

## 4 Method Details

### 4.1 F‐Type Pyocin Purification From *Pseudomonas Aeruginosa*

*P. aeruginosa* was cultured in 1.6 L of Luria‐Bertani (LB) medium (Tryptone 10 g/L, yeast extract 5 g/L, NaCl 10 g/L. from BD Biosciences, 214906) at 37°C with constant shaking at 220 rpm until the optical density at 600 nm (OD_600_) reached 0.5. To induce F‐type pyocin expression, mitomycin C (Aladdin, M476633) was added to the culture to a final concentration of 5 µg/mL, and induction was continued for 4 h. The bacterial culture was then subjected to ultrasonic lysis at 350 W for 5 min to ensure complete cell disruption. Cell debris was removed by centrifugation at 17 000 ×g for 1 h, and the resulting supernatant (containing F‐type pyocin) was filtered through a 0.22 µm filter. F‐type pyocin particles were pelleted from the filtered supernatant by batch ultracentrifugation at 150 000 ×g for 2 h.

The pelleted F‐type pyocin was resuspended in lysis buffer (25 mm Tris‐HCl buffer, pH 7.5, supplemented with 150 mm NaCl) and then layered onto a continuous 10%–40% (v/v) glycerol density gradient. Ultracentrifugation was carried out at 96 000 × g for 4 h. Large contaminants such as lipid droplets sedimented within the lower half of the gradient, whereas F‐type pyocin activity was enriched in the upper fraction. Accordingly, the upper half of the gradient was harvested and subjected to a second round of ultracentrifugation at 150 000 ×g for 4 h, and the resulting pellet was resuspended in lysis buffer. The resuspended sample was diluted to 70 mL with buffer QA (25 mm Tris‐HCl buffer, pH 7.5) and loaded onto an anion exchange chromatography column packed with Source 15Q resin (Cytiva, 17094701). Elution was performed using a linear gradient of 0%–100% 1 m NaCl in 25 mm Tris‐HCl (pH 7.5). Fractions containing highly pure F‐type pyocin were identified via negative staining transmission electron microscopy.

### 4.2 Bactericidal Assay

An overnight saturated culture of the indicator strain was diluted 1:50 (v/v) into pre‐warmed LB medium supplemented with 0.75% (w/v) agar (SolarBio, A8190). This mixture was poured into 10 cm culture dishes and allowed to solidify at room temperature to prepare indicator lawn plates. The F‐type‐pyocin‐containing test solution was serially diluted in lysis buffer, and aliquots of each dilution were spotted onto the surface of the indicator lawn plate. The plates were then incubated overnight in a 37°C incubator, after which zones of inhibition were observed and recorded.

### 4.3 Negative Staining

A 4 µL aliquot of the F‐type pyocin solution was applied to a hydrophilized carbon‐coated copper grid and incubated for 2 min. Excess solution was blotted away using filter paper, and the grid was stained three times with 4 µL aliquots of 0.2 g/L (w/v) uranyl acetate solution, with each staining step lasting 30–60 s. Residual uranyl acetate on the grid surface was thoroughly blotted off, and the grid was air‐dried and stored until subsequent imaging.

### 4.4 Cryo‐EM Sample Preparation and Data Acquisition

Cryo‐EM samples were prepared using a Vitrobot Mark IV (Thermo Fisher Scientific). F‐type pyocin purified from a 1.6 L *P. aeruginosa* culture was concentrated to a final volume of 30 µL using an ultrafiltration concentrator with a 100 kDa molecular weight cut‐off (Sartorius, VS0141). A 4 µL aliquot of the concentrated F‐type pyocin solution was applied to a hydrophilized Cu 300‐mesh Quantifoil R1.2/1.3 grid. The grid was incubated for 30 s at 8°C under 100% relative humidity, followed by blotting for 3.5 s and plunge‐freezing into liquid ethane. Frozen grids were subsequently transferred to liquid nitrogen for long‐term storage.

The quality of frozen grids was initially screened using a Tecnai Arctica 200 kV transmission electron microscope (Thermo Fisher Scientific). For grids exhibiting optimal sample distribution and particle quality, cryo‐EM data were acquired on a 300 kV Titan Krios microscope equipped with a Falcon 4 direct electron detector (Thermo Fisher Scientific). Data collection was performed at a counted pixel size of 0.926 Å, with each movie stack recorded at a total electron dose of ∼50 e^−^/Å^2^ and defocus values ranging from −1.5 to −2.5 µm. Automated data acquisition was carried out using EPU software (Thermo Fisher Scientific), and a total of 9,845 movies were collected in this study.

### 4.5 Image Processing and 3D Reconstruction

Beam‐induced motion correction was performed using MotionCor2 [42], and all subsequent image processing steps were conducted in cryoSPARC [43]. Patch‐based contrast transfer function (CTF) estimation was applied to each micrograph, and those with a CTF fit resolution worse than 4.5 Å were excluded from further analysis. Initial automated particle picking was performed using a blob picker, followed by preliminary 2D classification. 2D class averages displaying distinct features of the F‐type pyocin Cap or Tip regions were selected to generate templates for subsequent template‐based particle picking.

For the cap region, high‐quality particles were selected through multiple rounds of template‐based particle picking and 2D classification. An initial 3D reference model was generated via ab initio reconstruction under C1 symmetry. The selected 27 365 particles were subjected to multiple rounds of non‐uniform refinement with C6 symmetry imposed, resulting in a map with a global resolution of 2.55 Å.

For the tail tip region, high‐quality particles were similarly selected through multiple rounds of template‐based particle picking and 2D classification, with an initial 3D reference model generated by ab initio reconstruction in C1 symmetry. The 93 253 selected particles were processed via multiple rounds of non‐uniform refinement with C3 symmetry imposed, yielding a map with a global resolution of 2.29 Å.

For the FNIII‐AHS region, particle sub‐boxes were extracted with a downward shift based on the determined orientation of the Tail tip region. Following multiple rounds of 2D classification without recentering and particle re‐extraction, 49 990 high‐quality particles were obtained. An initial model was reconstructed based on the tip orientation, followed by 3D reconstruction with C3 symmetry imposed, resulting in a map with a resolution of 2.63 Å.

For the tail fiber region, particle sub‐boxes were extracted with a downward shift based on the determined orientation of the Tail tip region. After multiple rounds of 2D classification without recentering and particle re‐extraction, 20 186 high‐quality particles were isolated. An initial model was reconstructed based on the Tail tip orientation, followed by 3D reconstruction with C3 symmetry imposed, yielding a map with a resolution of 3.26 Å.

To further improve the resolution of the side fiber, C3 symmetry expansion was performed based on the Tail fiber orientation, and the particle center was shifted to the receptor‐binding domain (RBD) of a single side fiber. After verifying particle quality via 2 and 3D classification, C1 local refinement was conducted using a soft mask covering the RBD. This process yielded a map with a resolution of 2.78 Å from 60 558 particles.

### 4.6 Model Building and Structure Refinement

Initial atomic models of the cryo‐EM maps were constructed *de novo* using EMBuilder [44]. Iterative manual fitting and structural optimization were performed in Coot [45]. Real‐space refinement was accomplished using PHENIX [46]. All statistics for 3D reconstruction and model refinement are summarized in Table S1. Structural visualization and figure rendering of the final atomic models were generated with PyMOL [47] and UCSF ChimeraX [48].

### 4.7 Plasmid Construction and Pyocin Expression From *Escherichia Coli*

For plasmid pET‐pyocin^F1‐F18^, the gene cluster of *pyoF1* to *pyoF18* was amplified from *P. aeruginosa* PAO1 by polymerase chain reaction (PCR). The amplified gene cluster was ligated into the PCR‐linearized pET21 vector via Gibson assembly and transformed into *E. coli* DH5α strain. Single colonies with ampicillin resistance were selected and verified by DNA sequencing. The recombinant plasmid was then extracted and transformed into *E. coli* BL21(DE3) strain. For plasmid pET‐pyocin^TrP+F2‐F18^, it was constructed based on pET‐pyocin^F1‐F18^. The *TrP* gene was amplified from *P. aeruginosa* PAO1 by PCR, and the sequences of pET‐pyocin^F1‐F18^ except for the *pyoF1* open reading frame (ORF) were linearized by PCR. The *TrP* (or *alpD*) gene was ligated into the linearized vector to replace the *py oF1* ORF via Gibson assembly, followed by transformation into *E. coli* DH5α and BL21(DE3) strains as described above.

A saturated culture of *E. coli* BL21(DE3) harboring the corresponding plasmid was inoculated into 2 L of LB medium containing 50 µg/mL ampicillin (GPCSCI, AK052), and cultured at 37°C with shaking at 220 rpm until the OD_600_ reached 0.8. Isopropyl β‐D‐1‐thiogalactopyranoside (IPTG, from GPCSCI, AC367) was added to a final concentration of 0.5 mm, and the culture temperature was reduced to 18°C with shaking at 220 rpm for approximately 18 h. Bacterial cells were harvested by centrifugation at 4000 ×g for 15 min, resuspended in approximately 30 mL of lysis buffer (25 mm Tris‐HCl buffer, pH 7.5, supplemented with 150 mm NaCl), and then lysed by ultrasonication at 350 W for 10 min. Cell debris was removed by centrifugation at 23 000 ×g for 1 h. The supernatant was filtered through a 0.22 µm filter to obtain the crude pyocin extract, which could be diluted for use in bactericidal assays or negative staining observations.

## Author Contributions

Z.G., X.G. and J.W. conceived and designed the project. L.W. and L.M. contributed to sample preparation. Z.G. and Y.X. optimized cryo‐grid preparation and collected cryo‐EM data. Z.G. and X.G. processed the cryo‐EM data. J.W. built the atomic models. X.G. and J.W. drafted the manuscript. All authors reviewed, edited, and approved the final version of the manuscript.

## Conflicts of Interest

The authors declare no conflicts of interest.

## Supporting information




**Supporting file**: advs77063‐sup‐0001‐SuppMat.docx

## Data Availability

The data that support the findings of this study are openly available in wwPDB at http://www.rcsb.org, reference number 25VD, 25VF, 26CI, 25VJ, 25VE.
