# Supplementary material for: F‐Type Pyocin Versus Phage λ Tail: Conserved Hub, Divergent Fibers
Source: Adv Sci (Weinh). 2026 Aug 10:e77063. Online ahead of print. doi: 10.1002/advs.77063 (PMC13455828; doi:10.1002/advs.77063)
Supplement: Supplementary file 1 [file ADVS-9999-e77063-s001.docx]

Supplementary information for

**F-type pyocin versus phage λ tail: conserved hub, divergent fibers**

Supplementary Figures S1 to S8

Supplementary Table S1 to S2

# Supplementary Figures

Figure S1. **Cryo-EM processing workflow for the F-type pyocin.**
A. Schematic of the cryo-EM processing workflow.
B. Local resolution distribution of the cryo-EM maps[1].
C. Angular distribution plots of the cryo-EM particles.
D. Gold-standard Fourier Shell Correlation (FSC) curves[2].

Figure S2. **Functional role of the TrP in the F-type pyocin heterologously expressed in *E. coli*.**
A. Negative-stain electron micrographs of particles produced by expression of the pyocin gene locus *pyoF1*-*pyoF18* in *E. coli*.
B. Negative-stain electron micrographs of particles produced by expression of the *pyoF1*-*TrP* replacement construct in *E. coli*.
C. Spot titrations assay comparing the bactericidal activities of samples in panel A and panel B.

Figure S3. **Medium-resolution cryo-EM map showing the region spanning from the FNIII-2 domain to the side fiber.**

Figure S4. **Typical stacked ring counts of hexameric tail tube proteins.**

Figure S5. **Exploded view showing the structural assembly of the baseplate.**

Figure S6. **Three distinct structural arrangement of distal tail proteins (DTP) or tail tip middle proteins (TTMPs) and their associated side fiber complexes.**

Figure S7. **Homologue structures of the subdomains constituting the F-type pyocin side fiber.**

Figure S8. **AlphaFold-predicted C-terminal domain of PyoF10, PyoF11, and PyoF12, alongside a structural comparison with the C-terminus of phage λ gpJ.**

# Supplementary Tables

Table S1 **Cryo-EM data collection, refinement and validation statistics**

|  | Cap | Tail tip | FNIII-AHS | Tail fiber | Side fiber |
| --- | --- | --- | --- | --- | --- |
| Voltage (kV) | 300 | | | | |
| Electron exposure (e^-^/Å^2^) | 50 | | | | |
| Defocus range (μm) | -1.5～-2.5 | | | | |
| Pixel size (Å) | 0.926 | | | | |
| Micrographs (no.) | 9,845 | | | | |
| Symmetry imposed | C6 | C3 | C3 | C3 | C1 |
| Final particle images (no.) | 27,365 | 93,253 | 49,990 | 20,186 | 60,558 |
| Map resolution (Å) 0.143 FSC threshold | 2.55 | 2.29 | 2.63 | 3.26 | 2.78 |
| Map sharpening B factor (Å^2^) | -75.8 | -67.2 | -76.6 | -69.4 | -94.9 |
| CC (model vs. data) | 0.8652 | 0.8671 | 0.8003 | 0.7338 | 0.7789 |
| Model composition |  |  |  |  |  |
| Chain count | 18 | 24 | 3 | 12 | 3 |
| Non-hydrogen atoms | 21,570 | 39,204 | 11,319 | 26,916 | 5,736 |
| Protein residues | 2,844 | 5,094 | 1,476 | 3,588 | 765 |
| B factors (Å^2^) |  |  |  |  |  |
| Proteins | 36.10 | 34.13 | 22.79 | 188.18 | 83.12 |
| R.m.s. deviations |  |  |  |  |  |
| Bond lengths (Å) | 0.002 | 0.003 | 0.003 | 0.003 | 0.006 |
| Bond angles (°) | 0.509 | 0.549 | 0.567 | 0.581 | 0.717 |
| Validation |  |  |  |  |  |
| MolProbity score | 1.71 | 1.87 | 2.16 | 2.41 | 2.45 |
| Clashscore | 5.23 | 6.85 | 5.85 | 14.27 | 8.55 |
| Poor rotamers (%) | 3.39 | 2.51 | 3.47 | 3.48 | 4.84 |
| Ramachandran plot |  |  |  |  |  |
| Favored (%) | 98.22 | 96.93 | 93.78 | 95.17 | 92.62 |
| Allowed (%) | 1.78 | 2.83 | 5.81 | 4.80 | 7.38 |
| Disallowed (%) | 0.00 | 0.24 | 0.41 | 0.03 | 0.00 |
| PDB code | 25VD | 25VF | 26CI | 25VJ | 25VE |
| EMDB code | EMD-80401 | EMD-80403 | EMD-80538 | EMD-80404 | EMD-80402 |

Table S2 **Abbreviation and modeled residues of F2-type pyocin in this work**

| **F2-type pyocin protein** | **Conserved feature** | **Abbreviation** | **Length** | **Modeled residues in this work** |
| --- | --- | --- | --- | --- |
| AlpD | Tail terminator protein | TrP | 151 | 4-151 |
| PyoF2 | Tail tube protein | TTP | 164 | 2-164 |
| PyoF3 | Tail assembly chaperone | TAC | 115 | - |
| PyoF4 |  |  | 84 | - |
| PyoF5 | Tape measure protein | TMP | 612 | 579-672 |
| PyoF6 | Distal tail protein | DTP | 113 | 3-113 |
| PyoF7 | Baseplate hub protein 1 | BHP1 | 231 | 1-232 |
| PyoF8 | Putative tail assembly protein | - | 256 | - |
| PyoF9 | Tail assembly protein | TAP | 200 | 131-200 |
| PyoF10 | Baseplate hub protein 2 | BHP2 | 1204 | 4-819 |
| PyoF11 | Putative receptor binding protein | - | 97 | - |
| PyoF12 | Putative receptor binding protein | - | 262 | - |
| PyoF13 | Side fiber | - | 363 | 2-363 |
| PyoF14 | Side fiber-associated protein | - | 111 | - |
| PyoF15 | Side fiber-associated protein | - | 76 | - |
| PyoF16 | - | - | 350 | - |
| PyoF17 | - |  | 100 | - |
| PyoF18 | - |  | 76 | - |

**References**

[1]	A. Kucukelbir, F. J. Sigworth, H. D. Tagare, *Nat Methods* **2014**, *11* (1), 63, https://doi.org/10.1038/nmeth.2727.

[2]	P. B. Rosenthal, R. Henderson, *J Mol Biol* **2003**, *333* (4), 721, https://doi.org/10.1016/j.jmb.2003.07.013.
